# Risk stratification for early and late falls in acute care settings

**DOI:** 10.1111/jocn.16267

**Published:** 2022-02-27

**Authors:** Masae Satoh, Takeshi Miura, Tomoko Shimada, Toyoko Hamazaki

**Affiliations:** ^1^ 13155 Department of Nursing Graduate School of Medicine Yokohama City University Yokohama Japan; ^2^ Nursing Department Yokohama City University Hospital Yokohama City University Yokohama Japan

**Keywords:** acute care setting, early falls, late falls, risk factors, risk prediction

## Abstract

**Background and Aims:**

Falling generally injures patients, lengthens hospital stays and leads to the wastage of financial and medical resources. Although falls can occur at any stage after hospital admission, there are no studies that characterise falls with length of hospital stay in acute care settings. This study aims to clarify risk stratification of early and late falls in acute care settings.

**Methods:**

A retrospective cohort study was conducted for participants who were admitted to a teaching hospital in Japan. Patients’ falls were divided into two groups based on the median of the fall date (day 10). Considering a 70/30 split, the logistic regression model was used to extract independent predictors for early and late falls for nine risk variables based on exploratory analysis among 26 items selected from the modified Japanese Nursing Association Fall Risk Assessment Tool, and risk models were validated. This study was conducted according to the STROBE guideline.

**Results:**

Of the 10,975 patients admitted, 87 and 90 with early and late falls, respectively, were identified. The five significant risk factors extracted for early falls were fall history, muscle weakness, impaired understanding, use of psychotropics and the personality trait of ‘doing everything on one's own’; risk factors identified for late falls were being older than 65 years, impaired extremities and unstable gait, in addition to muscle weakness. Using these variables for early and late falls in the validation cohort, the concordance indices of the risk models were both over 0.80.

**Conclusions:**

By separately extracting risk factors for early and late falls in an acute care hospital setting, this study shed light on the characteristics of the respective types of falls.

**Relevant to clinical practice:**

As the risk factors of falls vary according to the length of hospitalisation, specific preventive care can be implemented to avoid fall incidents.

**What does this paper contribute to the wider global clinical community?**

- Although high fall rates are observed during the early period of stay in various settings of nursing care, risk stratification of early and late falls has never been undertaken in acute care settings.
- This original research article describes sequential changes in risk factors for falls during hospitalisation in acute care setting.
- The common risk factors for early and late falls and specific risk factors for each of them were identified for the implementation of preventive care during hospitalisation.

## 1 BACKGROUND

Falls among older patients are one of the most frequently occurring complications experienced in the wards (Bouldin et al., 2013). A significant percentage of such falls results in serious injuries and is a major cause of older people's prolonged hospital stays (Miake‐Lye et al., 2013). In addition, the prospective descriptive study of inpatient falls showed that falls in the hospital affect young and older patients (Hitcho et al., 2004). The medical costs associated with such falls are significant and increase the overall costs of healthcare (Heinrich et al., 2010). Prevention of falls is a global concern, and one of the patient safety goals of the Joint Commission International states that its prevention ‘reduces the risk of patient injury from falls’ (Joint Commission International, 2017).

In acute care settings, the commonly used fall risk assessment tools are as follows: the St. Thomas Risk Assessment Tool in Falling Elderly Inpatients (Oliver et al., 1997), Morse Fall Scale (Morse et al., 1989) and Hendrich II Fall Risk Model (Hendrich et al., 2003), which are also nursing assessment scales that take less than a minute for assessment. For example, the St. Thomas Risk Assessment Tool in Falling Elderly Inpatients includes the following categories: (1) history of falls, (2) mental states, (3) visual impairment, (4) frequent toileting, and (5) transfer and mobility (Oliver et al., 1997). The Morse Fall Scale includes the following domain categories: (1) history of falls, (2) secondary diagnosis, (3) ambulatory aids, (4) intravenous (IV) saline lock, (5) gait and (6) mental state. The Hendrich II Fall Risk Model includes the following categories: (1) confusion/disorientation, (2) depression, (3) altered elimination, (4) dizziness/vertigo, (5) gender, (6) administration of antiepileptics/benzodiazepines and (7) get‐up‐and‐go test/ability to rise in a single movement. These tools are intended for use by nurses at the point of care to predict patients’ risk of falling. Items on the scale are numerically scored, and scores greater than the cut‐off points are deemed as a high risk for falls. These tools, which comprise the predictive validity criteria for fall risk assessment tools in clinical practice as suggested by Oliver et al. (2004) were evaluated in multiple hospitals, and their sensitivity and specificity were both found to be greater than 70%.

However, in Japan or Asian countries, these models were not as good as those evaluated in European countries, and some studies discussed the possibility of differences in fall risks between Western and Asian populations (Chow et al., 2007; Kim et al., 2007; Toyabe, 2010). Japan uses the Japanese Nursing Association Fall Risk Assessment Tool, which is modified in each hospital for optimal application. It contains items related to the treatment stage, patients’ personalities and experiences in the hospital environment. These modified factors are not included in the three common risk assessment tools mentioned above. Higaonna (2015) showed that the modified Japanese Nursing Association Fall Risk Assessment Tool demonstrated good predictive validity in a Japanese university hospital.

Unfortunately, these scales do not encompass all the intrinsic and extrinsic fall risk factors identified as causative factors for inpatient falls. The assessment tool contains more than 30 risk variables, and predictive validity (estimated as area under the ROC curve) decreased in accordance with the length of hospital stay. Hence, to incorporate the causative factors, new or modified assessment strategies should be considered (Spoelstra et al., 2012). To date, risk prediction models have generally been designed for all hospitalised falls. It is necessary to recognise that falls, especially injurious falls, occur during the relatively early period of hospital stay. Cho et al. (2020) reported that 52% of falls occurred within 10 days of admission, and Higaonna (2015) reported that 40.3% occurred during the first week of the 28 days of observation. Recently, Francis‐Coad et al. (2020) reported that for injurious falls in acute medical and surgical units, the peak occurrence time was between day 1 and day 4 with 46.8% having occurred by the third day of admission. These circumstances raise the question of whether risk assessment of falls should be developed as a single cohort for all hospitalised patients or not.

If risk factors specific to early or late falls, or those common to both, could be predicted, it would be possible to take reasonable precautions to safeguard potential falls during the course of patients’ hospital stay. Falls occurring by day 10 of the hospital stay (i.e. before the median time of all falls) were defined as early falls, whereas those occurring later than day 10 were defined as late falls. This study, therefore, aimed to shed light on the characteristics of early and late falls in acute care settings and separately stratify their respective risk factors.

## 2 METHODS

### 2.1 Study design and setting

This was a retrospective cohort study that used medical records from a University Hospital (acute phase, special function hospital) in Japan, having 612 general beds and reporting 2,04,000 inpatients annually. The survey was conducted between 1 October 2018 and 30 September 2019, and the selected venue had an average bed utilisation rate of 85.2% in 2019. This study was conducted according to the Strengthening the Reporting of Observational Studies in Epidemiology (STROBE) guideline for reporting case‐control studies (Appendix S1).

### 2.2 Participants

**FIGURE 1:**
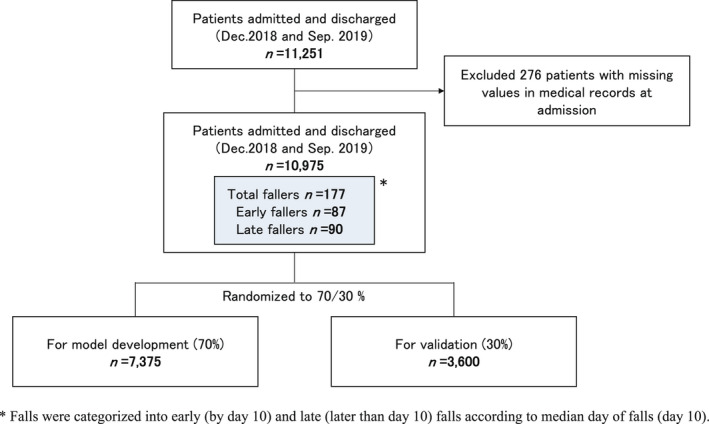
Flow chart to create the data set and test set [Colour figure can be viewed at wileyonlinelibrary.com]

This study's participants comprised 11,251 inpatients aged 16 years or older, who were considered old enough to make an informed consent to participate, and were admitted to general wards and discharged during the survey period. The study did not include inpatients in the intensive care unit or the psychiatric, paediatric and infectious disease wards, where special care was given to patients (Figure 1). Only patients without missing values, confirmed in the records at admission were included in the analysis (Figure 1).

### 2.3 Fall risk assessment tool

This study used the modified version of the Japanese Nursing Association Fall Risk Assessment Tool, which originally consisted of nine categories and 32 items when it was introduced in 1999. This is the most commonly used scale for assessing fall risks in Japanese hospitals, with some modifications in each institute. Higaonna’s (2015) study among patients 15 years and older in a Japanese university hospital showed that it had good predictive validity.

The original version used in this study was modified to six categories and 26 items by the hospital's risk management committee based on submitted incident reports and clinical expertise. The accuracy of data input was ensured by a method confirmed by leader nurses and nurses in‐charge of support. When nurses were inexperienced or unfamiliar with the variables and uncertain about their definitions, especially when it was better to share questions/answers among themselves to guarantee reliability and validity, they held discussions among the evaluators. It was previously reported that more than two variables from among the six selected by multiple logistic regression analysis showed high risk of patients, with 71.8% sensitivity and 77.2% specificity, which were considered to indicate sufficient accuracy for a fall assessment tool (Miura & Satoh, 2021).

The sample size was estimated using G*Power statistics (Faul et al., 2009) for 26 predictors with an effect size f^2^ of 0.15 which is recommended by Cohen's (1988, 1992) guidelines as the medium effect size—and power of 0.8, calculated as 175 falls.

The 26 items were categorised into the following 10 categories: age, fall history, sensory/motor functions, mobility, cognition, medication, elimination, diagnosis and treatment stage, personality and hospitalisation (Table 1). The individual items were as follows:

- 65 years old or older
- A history of falls
- Visual impairment
- Impaired extremities
- Muscle weakness
- Using mobility assistive devices
- Requiring mobility assistance
- Unstable gait
- Impaired understanding
- Taking analgesics
- Taking narcotics
- Taking psychotropic medication
- Taking antihypertensive medication
- Taking diuretics/laxatives
- Having urinary and faecal incontinence
- Frequent toilet use
- Toileting more than twice per night
- Anaemia
- Pretibial oedema
- Central nervous system disease
- Being within three days after surgery
- In rehabilitation
- IV lines or tubes inserted
- Hesitating to use the call light
- Doing everything on one's own
- New to the hospital environment

**TABLE 1:** Prevalence and Wilcoxon rank‐sum test for each variable between fallers and non‐fallers

Variable category	Variables	Total		Non‐fallers		Fallers		Pearson's	Wilcoxon
		*n* = 10,975		*n* = 10,798		*n* = 177		chi‐square	rank‐sum
		*n*	(%)	*n*	(%)	*n*	(%)	test *p*	test *p*
Gender	Male	5,789	(52.7)	5,690	(52.7)	99	(55.9)	0.392	0.533
Surgery	Yes	4,354	(39.7)	4,301	(39.8)	53	(29.9)	0.008	0.184
Age (1)	>65	5,832	(53.1)	5,696	(52.8)	136	(76.8)	<0.001	0.591
Fall history (1)	Fall history	980	(8.9)	927	(8.6)	53	(29.9)	<0.001	0.203
Sensory function (1)	Visual impairment	1,108	(10.1)	1,082	(10.0)	26	(14.7)	0.041	0.873
Motor function (1)	Impaired extremities	792	(7.2)	757	(7.0)	35	(19.8)	<0.001	0.022
Mobility (4)	Muscle weakness	1,745	(15.9)	1,647	(15.3)	98	(55.4)	<0.001	0.898
	using mobility assistive devices	1,834	(16.7)	1,744	(16.2)	90	(50.8)	<0.001	0.967
	Requiring mobility assistance	1,492	(13.6)	1,402	(13.0)	90	(50.8)	<0.001	0.243
	Unstable gait	595	(5.4)	549	(5.1)	46	(26.0)	<0.001	0.782
Cognition (1)	Impaired understanding	510	(4.6)	475	(4.4)	35	(19.8)	<0.001	0.001
Medication (5)	Analgesics	1,412	(12.9)	1,370	(12.7)	42	(23.7)	<0.001	0.945
	Narcotics	206	(1.9)	197	(1.8)	9	(5.1)	0.002	0.610
	Psychotropics	1,220	(11.1)	1,177	(10.9)	43	(24.3)	<0.001	0.704
	Antihypertensive	3,009	(27.4)	2,940	(27.2)	69	(39.0)	0.001	0.850
	laxatives/diuretics	1,416	(12.9)	1,373	(12.7)	43	(24.3)	<0.001	0.826
Elimination (3)	Urinary/bowel incontinence	266	(2.4)	249	(2.3)	17	(9.6)	<0.001	0.570
	frequent toileting	211	(1.9)	205	(1.9)	6	(3.4)	0.152	0.202
	toileting >2x per night	5,681	(51.8)	5,574	(51.6)	107	(60.5)	0.020	0.505
Diagnosis (3)	Anaemia	678	(6.2)	654	(6.1)	24	(13.6)	<0.001	0.156
	Pretibial oedema	310	(2.8)	300	(2.8)	10	(5.6)	0.022	0.005
	CNS disorder	552	(5.0)	523	(4.8)	29	(16.4)	<0.001	0.440
Treatment stage (3)	Within 3 days postop	42	(0.4)	42	(0.4)	0	(0.0)	1.000^†^	NA
	Rehabilitation	67	(0.6)	62	(0.6)	5	(2.8)	0.004^†^	0.983
	IV lines or tubes	2,280	(20.8)	2,220	(20.6)	60	(33.9)	<0.001	0.637
Personality (2)	‘Hesitate to use call light’	153	(1.4)	141	(1.3)	12	(6.8)	<0.001	0.042
	‘Do everything on one's own’	301	(2.7)	284	(2.6)	17	(9.6)	<0.001	0.003
Hospitalisation (1)	New to the hospital environment	7,588	(69.1)	7,470	(69.2)	118	(66.7)	0.473	0.167

Abbreviations

^†^, *p* value of Fisher's exact test; NA, not applicable.

The patients’ fall risk assessments were performed by registered nurses in the ward at the time of admission and recorded in an electronic charting system.

### 2.4 Fall definition and identification

As per the Frailty and Injuries Cooperative Studies of Intervention Techniques trial, a fall is defined as a state in which a body part, other than the sole, touches the floor (Buchner et al., 1993). Inpatients who fell (the ‘fall group’) were identified from the electronic medical records that contained details of all incidents of falls, as described by the medical staff who had found the inpatients who had fallen. Patients with no description of falls were included in the ‘no fall’ group. This process was conducted by the staff in the medical information department.

### 2.5 Data collection

Based on inpatient administrative records, fall risk assessment results were combined to create a database for analysis. Data regarding age, sex, admission and discharge dates and clinical information were obtained from these records.

### 2.6 Statistical analysis

Statistical analysis was performed using JMP^®️^ Pro15 (SAS Institute Inc., Cary, NC, USA) and SPSS^®^□ Statistics Version 26.0. Patients’ characteristics and each of their risk variables were examined using Pearson's chi‐square test or Fisher's exact test, if the number of falls was smaller than five. The statistical significance was set at *p* < 0.05. The Kaplan‐Meier method was used to estimate time to fall using a Wilcoxon rank‐sum test. The statistical significance was set at *p* < .05.

#### 2.6.1 Exploratory analysis to select significant and time‐dependent predictors for all falls

Logistic regression models were used to examine the effect of characteristic risk factors for all falls using backward stepwise selection of predictors, with *p* > .05 for exclusion.

The 11 statistically significant variables obtained by the Kaplan‐Meier fall probability analysis and the logistic regression analysis were combined (see Table 2) and used as factors for corresponding multivariable logistic models in the analyses.

**TABLE 2:** Sequential change in odds ratios of the variables showing significance for falls during the course of hospital stay

Day of stay	Day1	≤day2	≤day3	≤day4	≤day5	≤day6	≤day7	≤day8	≤day9	≤day10	≤day14	≤day28	All cases
*N*	7	20	31	43	54	62	69	78	87	97	121	153	177
Age > 65									1.80	1.74	1.86	2.04	1.92
									(1.07–3.05)	(1.06–2.85)	(1.20–2.91)	(1.37–3.05)	(1.33–2.78)
Fall history								1.81	2.10	1.77		1.57	1.48
								(1.06–3.10)	(1.27–3.46)	(1.09–2.86)		(1.06–2.34)	(1.02–2.16)
Impaired extremities												1.86	1.98
												(1.20–2.88)	(1.32–2.96)
Muscle weakness					2.13	2.21	2.13	2.87	2.69	2.46	2.53	2.30	2.45
					(1.04–4.35)	(1.13–4.29)	(1.14–3.99)	(1.60–5.17)	(1.54–4.71)	(1.45–4.19)	(1.58–4.05)	(1.51–3.49)	(1.66–3.61)
Requiring mobility assistance		6.18	4.80	2.87	2.82	2.53	2.36		1.98	2.34	2.31	2.05	2.01
		(1.81–21.14)	(1.69–13.64)	(1.20–6.90)	(1.31–6.07)	(1.25–5.15)	(1.21–4.63)		(1.09–3.57)	(1.22–3.50)	(1.41–3.79)	(1.32–3.18)	(1.34–3.02)
Unstable gait				2.24							1.81	1.96	2.30
				(1.04–4.79)							(1.11–2.96)	(1.25–3.05)	(1.53–3.45)
Impaired understanding		5.43	3.08	2.92	2.41	2.37	2.01						
		(1.70–17.40)	(1.20–7.91)	(1.31–6.52)	(1.16–5.02)	(1.04–3.41)	(1.03–3.95)						
Psychotropi cs						1.88	2.16	2.28	2.06	1.87	2.11	2.11	1.93
						(1.04–3.41)	(1.25–3.75)	(1.37–3.82)	(1.25–3.39)	(1.15–3.03)	(1.38–3.22)	(1.45–3.10)	(1.34–2.78)
‘Do everything on one's own’			2.80	2.42	2.73	2.29							
			(1.08–7.26)	(1.02–5.72)	(1.26–5.93)	(1.07–4.87)							

Notes

The eleven risk variables which were selected by stepwise regression model for all falls and by Wilcoxon rank‐sum test were evaluated in multivariate regression analysis at each point of hospital stay. The odds ratios showing statistically significant (*p* < 0.05) were selected and described in respective points (see details in Materials and Methods).

The effects of time‐dependent risk factors on falls during hospital stay were sequentially investigated. Thus, multiple logistic regression analysis was performed for falls that occurred from day 1 to day 10, at day 14, at day 28 and all falls (see Table 2). At each point of analysis, the falls at each time point were excluded from the initial non‐fall population to arrive at the final non‐fall population.

#### 2.6.2 Risk stratification relating to early and late falls

Falls occurring by day 10, that is before the median time of all falls, were defined as early falls, whereas those occurring later than day 10 were defined as late falls. For the analysis of each primary outcome, data of early and late falls were randomly divided into two subsets with a split of 70/30, with the former and latter being used for model development and validation, respectively (Figure 1). Two sets of multiple logistic models for early and late falls were constructed as a data set for development, using the same variables obtained by exploratory analysis.

For early and late falls, receiver operating characteristic curves—a graphical plot of a test's true positive rate (sensitivity) versus its false‐positive rate (specificity)—were created for the validation data set. Each point on the curve indicates a pair of false positive and true positive rates that are achieved using a particular threshold to dichotomise the predicted probabilities. To evaluate the performance of the models, the concordance index (C‐index: a measure of model discrimination), which is the area under the receiver operating characteristic curve, was calculated for each validation set using the bootstrap method with 1,000 random samples.

### 2.7 Ethical considerations

The Ethics Committee of Epidemiological Research of Yokohama City University approved the study (no. B201000047), which was conducted in accordance with the Declaration of Helsinki. An information disclosure document outlining the research and its use was posted on the hospital's website. This ensured that participants had the opportunity to receive research information and decide whether or not to use it. Patient data were anonymised using a correspondence table and strictly managed with a password assigned to the server of the target facility. After digitising the questionnaires, all the originals were shredded.

## 3 RESULTS

After excluding 276 patients with missing values, confirmed in the records at admission; finally, 10,975 patients were included in the analysis (Figure 1). One hundred and seventy‐seven first falls were identified from among 210 falls in total. The median lengths of stay in the fall and non‐fall groups were 22 days and 7 days, respectively, showing a significant prolongation of hospital stay (*p* < .0001). The incidences of falls indicated that they occurred as early as the day of admission, with their rate peaking at day 2, and gradually decreasing thereafter (Supporting Information Figure S1). Day 10 was considered as the median day of falls.

Except for the variables of frequent toileting and new to the hospital environment, and being within three days of surgery, all the other 23 risk variables showed significant differences in their prevalence between fallers and non‐fallers (Table 1).

**FIGURE 2:**
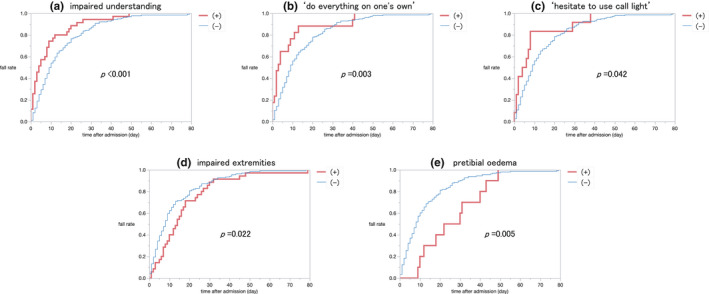
Kaplan‐Meier curve of falls, with or without the respective risk variables. Occurrence of fall according to the respective risk variables was plotted using the Kaplan‐Meier method. The risk variables showing significant differences by Wilcoxon rank‐sum test are shown [Colour figure can be viewed at wileyonlinelibrary.com]

The Kaplan‐Meier probability method was used to estimate time to fall with a Wilcoxon rank‐sum test (Table 1). A significant difference was noted in five variables: ‘impaired understanding’, ‘hesitancy to use the call light’, ‘doing everything on one's own’, ‘impaired extremities’ and ‘pretibial oedema’. When the Kaplan‐Meir curve was created (Figure 2), fallers described as having impaired understanding, hesitancy to use the call lights and ‘doing everything on their own’ were found to fall earlier than those described as having impaired extremities or pretibial oedema, who were found to fall later. This trend suggested that these variables could be factors relevant to early or late falls.

Following the exploratory analysis, the significant risk factors for all cases included seven variables: age > 65 years, fall history, impaired extremities, muscle weakness, requiring mobility assistance, unstable gait and use of psychotropics (see Table 2).

The five variables obtained through the Kaplan‐Meier analysis and the seven obtained through exploratory analysis (with ‘impaired extremities’ being common) were combined and applied as factors for multiple logistic regression analysis.

### 3.1 Time‐dependent predictors for falls

The results of the effect of time‐dependent significant risk factors for falls during the course of hospital stay based on sequential changes in their respective odds ratios are summarised in Table 2. Nine variables were found to be significant for falls of either type, but interestingly, among them, impaired understanding and doing everything on one's own were only found during the early phase (by day 7); requiring mobility assistance, muscle weakness and use of psychotropics were found throughout the length of hospital stay; and fall history, age > 65 years and impaired extremities were found later than day 8. Unstable gait was mostly observed in the later phase, besides a few cases in the early phase.

### 3.2 Risk stratification of early and late falls

Two risk models were developed, and multiple logistic regression analysis was separately performed for early falls (*n* = 87) and late falls (*n* = 90) using the nine variables. The final logistic models with odds ratios and 95% confidence intervals are shown in Table 3. The two groups shared an overlapping variable, that is, muscle weakness, which was characterised as mobility. The independent significant variables for early falls were as follows: a history of falls, impaired understanding, taking psychotropic medications and the personality trait of ‘doing everything on one's own’, whereas for late falls the variables were being over 65 years of age, impaired extremities and an unstable gait.

**TABLE 3:** Risk models for early and late falls

Variable category	Variables	Early falls (n=87)^†^	Late falls (n=90)^‡^
		OR (95% CI)	OR (95% CI)
Age	>65	1.64(0.87–3.10)	1.88(1.04–3.39)*
History	Fall history	2.10(1.16–3.79)**	1.04(0.54–1.99)
Motor function	Impaired extremities	1.25(0.59–2.64)	2.58(1.40–4.76)**
Mobility	Muscle weakness	3.25(1.69–6.24)**	2.50(1.36–4.61)**
	requiring mobility assistance	1.73(0.88–3.40)	1.81(0.95–3.43)
	unstable gait	1.29(0.64–2.60)	2.88(1.49–5.54)**
Cognition	Impaired understanding	2.00(1.03–3.87)*	0.72(0.30–1.70)
Medication	Psychotropics	2.34(1.33–4.11)**	1.73(0.96–3.12)
Personality	‘Do everything on one's own’	2.71(1.29–5.67)**	0.74(0.22–2.49)

Note

OR, odds ratio; 95% CI, 95% confidence interval.

† C‐index of development cohort 0.830, C‐index of validation cohort (95% CI) 0.816(0.719–0.885).

‡ C‐index of development cohort 0.801, C‐index of validation cohort (95% CI) 0.868(0.781–0.926).

** *p*<0.01

* *p*<0.05.

### 3.3 Model performance

**FIGURE 3:**
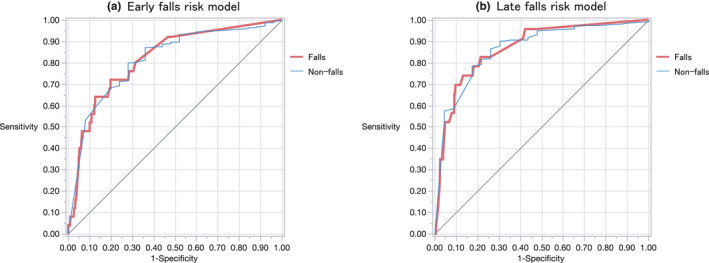
Receiver operating characteristic curves for early and late falls in a validation data set. The AUC was 0.816 for the early falls [95% confidence interval (CI), 0.72–0.89] and 0.868 for the late falls [95%CI, 0.78–0.93] [Colour figure can be viewed at wileyonlinelibrary.com]

The areas under the receiver operating characteristic curves for both models (Figure 3) and its calibration across risk groups were evaluated to determine the performance. In the development cohort, the C‐indices of early and late falls were 0.830 and 0.801, respectively, whereas in the validation cohort, for early falls, they were 0.816, 95% CI (0.719–0.885), and for late falls they were 0.868, 95% CI (0.781–0.926).

## 4 DISCUSSION

### 4.1 Summary output: Predictive validities of risk models created for early and late falls in an acute phase hospital

This study was undertaken in an acute phase hospital setting to shed light on early and late falls and characterise them based on risk assessment. The risk models were separately constructed for each subgroup and found to be reasonably validated in the test datasets. Five significant risk factors: fall history, muscle weakness, impaired understanding, use of psychotropics and the personality trait of ‘doing everything on one's own’ were extracted for early falls. For late falls, in addition to muscle weakness, the risk factors extracted were being over 65 years of age, impaired extremities and an unstable gait. As stated previously, the receiver operating characteristic of the risk models for early and late falls in the validation cohorts were 0.816 and 0.868, respectively.

To the best of our knowledge, this is the first study to report on risk variables for early and late falls in an acute phase hospital setting and to categorise the variables as common/reversible, specifically in terms of the period of hospital stay.

### 4.2 Significant predictors for all falls among the 26 variables of the modified Japanese Nursing Association Risk Assessment Tool

Fall assessment tools should be simple, without too many items, easy to use and predictable without complicated calculations, especially in acute care hospitals (Wyatt & Altman, 1995), so as to minimise the time and burden required for this work (Perell et al., 2001). As the modified Japanese Nursing Association Fall Risk Assessment Tool used in this study included 26 variables, it would perhaps be necessary to compress the number of variables to predict falls for clinical practice. For the exploratory analysis, seven significant predictors—age > 65 years, fall history, impaired extremities, muscle weakness, requiring mobility assistance, unstable gait, impaired understanding and medication of psychotropics—were extracted for all falls using multiple logistic analysis, through backward stepwise selection. Most of these variables had been included by studies using other risk assessment tools in acute care settings (Hendrich et al., 2003; Morse et al., 1989; Myers, 2003; Oliver et al., 2004; Perell et al., 2001). It is easy and useful for nurses to choose seven variables from among 26, without losing clinically meaningful predictability. However, when ‘sequentially’ looking at risk variables for falls in the course of hospital stay, it was interesting to find hospital stay‐dependent risk predictors (Figure 2 and Table 2).

### 4.3 Hospital stay‐dependent risk predictors for early and late falls

The risk models for the predictivity of falls have been reported to change during the course of hospitalisation in acute care settings (Higaonna, 2015), subacute care settings (Rapp et al., 2016; Vassallo et al., 2003) or during the follow‐up period for Parkinson's disease (Duncan et al., 2012), which may be related to changes in intrinsic patient factors or other external factors over the course of time. This study looked at candidate factors for early and late falls using the Kaplan‐Meier curve. The Kaplan‐Meier fall probability method was used to estimate the time of falls with a Wilcoxon rank‐sum test (Table 1). The Kaplan‐Meier fall curve clearly showed that the timing of falls during hospitalisation differed with or without the presence of certain variables (Figure 2). Patients having impaired understanding and those with the personality traits of ‘doing everything on their own’ and ‘hesitancy to use the call light’ tended to fall earlier than those not having these traits. These variables seemed to be relevant to some characteristics of older adults taking risks and communication failures between patients and medical staff, as found in a previous study by Haines et al. (2015) that showed the key factors influencing risk‐taking behaviour as follows: (i) the risk compensation ability of older patients, (ii) their willingness to ask for help, (iii) desire to test their physical boundaries, (iv) communicate about failure among older adults, informal caregivers and health professionals, and (v) delayed provision of help. For preventing potential falls in patients, it is important for nurses to keep in mind the above factors.

In contrast, falls occurred later in patients with impaired extremities and pretibial oedema than those not having these symptoms. These variables are disease‐specific factors apart from mental status, which reasonably could be assumed to be controlled in the early phase of hospitalisation in this study. These time‐dependent characteristics of fallers were visualised by considering each variable's odds ratios sequentially, from day 2 to day 28 (Table 2). It is interesting to note that significant predictors were time‐dependent, as previously found in subacute settings (Rapp et al., 2016). Thus, some predictors, such as impaired understanding and possessing the personality trait of ‘doing everything on one's own’ were found only during early hospitalisation (up to day 7); variables such as age >65 years and impaired extremities were found during late hospitalisation (day 9 onwards), whereas some others were found throughout the course of hospitalisation. The early risk factors observed in the Kaplan‐Meier fall probability analysis (impaired understanding and ‘doing everything on one's own’) were extracted as significant risk factors from day 2 to day 7. Amongst the late risk factors observed in the Kaplan‐Meier fall probability method, impaired extremities were extracted as a significant risk factor on day 28.

### 4.4 Risk models for early and late falls

For further characterisation and evaluation of predictive variables for early and late falls, risk models that could be validated were created. Based on their median time, falls were classified into early and late falls—87 and 90, respectively. The data set was also randomly divided as 70/30 for deprivation and validation. The validation step used the bootstrap method (Efron, 1979), in which, independent, random samples (size *N*) are repeatedly drawn (*n* = 1000), with replacements from an original data set of size *N*. The empirical distributions can in turn be used to estimate the standard errors for these statistics, and the confidence intervals for the theoretical parameters that these statistics estimate. Our models for early and late falls showed good predictions with C‐indices over 0.8.

The models were created by using nine significant variables (shown in Table 2). The risk models for early falls included five significant risk factors: fall history, muscle weakness, impaired understanding, psychotropics and personal trait of’doing everything on one's own’, whereas the one for late fallers included four significant risk factors: age > 65, impaired extremities, muscle weakness and unstable gait. This was consistent with the results of the hospital‐stay‐dependent risk factor analysis (Table 2).

Having considered the fact that only those who stayed in the hospital for over 10 days could be classified as late fallers, the risk factors of late fallers (*n* = 90) from among the patients who stayed in hospital over 10 days (*n* = 4010), excluding patients who were discharged by day 10, were analysed (Supporting Information Table S1 and Figure S2). Multiple logistic regression analysis was used to extract significant risk variables similar to those in Table 3, except psychotropics, which was included as a significant variable in this analysis. Consequently, the main context of observation was consistent with risk factors predicted at admission for late falls. These results suggest that risk factors obtained for late falls (Table 2) were reproducible and clinically meaningful.

### 4.5 Importance of identifying risk factors for falls instead of categorising low‐ or high‐risk groups

Fall risk assessment tools commonly used in acute care settings were set up to identify high‐risk patients from the group of patients with low risk. Sensitivity and specificity values >0.7 were considered to have enough accuracy as a fall assessment tool (Oliver et al., 2004). However, previous research demonstrated that risk scores do not reliably predict which patients are at risk of falls/injurious falls (Healey & Haines, 2013; Mion et al., 2012). Francis‐Coad et al. (2020) investigated the association between the characteristics of injurious falls along with fall preventive interventions in acute care settings. They reported that risk scores are not useful for providing fall management, given that nearly half of the cohort was classified as having a low risk of falls; moreover, patients are more likely to undergo fall interventions when they are identified as high risk and away from patient‐centred programmes that focus on the risk factors of each patient. This study could only identify predictors of early falls that could be differentiated from late falls and analyse those factors and create strategies by focusing on specific predictors.

### 4.6 Reasons for the different risk factors between early and late falls

Risk factors and risk assessment tools for falls among inpatients were systemically reviewed by Oliver et al. (2004), who showed that despite the heterogeneity of settings, a few significant fall risk factors consistently emerged: gait instability, agitated confusion, urinary incontinence/frequency, history of falls and prescriptions of sedatives/hypnotics. In this study in an acute care setting, the risk factors for early and late falls were in contrast. For early falls the risk factors were a history of falls, muscle weakness, impaired understanding, using psychotropics medication and the personality trait of ‘doing everything on one's own’. These factors (except muscle weakness and history of falls) were categorised mainly as mental states—cognition, medication having an effect on the central nervous system and personality traits—which are all relevant factors hampering the maintenance of mutual communication between patients and medical staff (Haines et al., 2015).

With regard to the personality trait of ‘doing everything on one's own’, it might be reminiscent of the findings of a classic qualitative study on falls in an acute care facility during the late 1980s and early 1990s by Chenitz et al. (1991), who summarised the characteristics of fallers based on qualitative analyses. They found some patients who deliberately ‘take a risk’ in an attempt to regain or test the ability to walk. Their findings implied that those who anticipated a short stay or were less ill were more likely to maintain self‐dependence, which could possibly explain why they were prone to early falls.

When considering whether mental state was a risk variable in the three commonly used methods—St. Thomas Risk Assessment Tool in Falling Elderly Inpatients (Oliver et al., 2004), Morse Fall Scale (Morse et al., 1989) and Hendrich II Fall Risk Model (Hendrich et al., 2003, 2020)—for assessing patients’ likelihood of falling in an acute care setting, it was found that all the three tools included this variable in their assessment. The St. Thomas Risk Assessment Tool in Falling Elderly Inpatients showed that ‘agitation’ had a higher odds ratio than ‘falls as the presenting complaint’ and ‘unstable gait’. Furthermore, the Morse Fall Scale had the variables of ‘forgets limitations’ versus ‘oriented to own ability’ for mental states. Finally, the Hendrich II Fall Risk Model had confusion/disorientation as a risk variable, which was consistent with the findings by Ivziku et al. (2011), who evaluated the predictive validity and inter‐rater reliability of this model in an Italian university hospital and showed that its confusion and depression factors were strongly associated with falls. Although all the three models had mental states or characteristics of patients as risk variables, this study demonstrated that this factor could be a significant risk variable specific to early falls in acute care settings.

In contrast to the variables for early fallers, the ones for late fallers were age >65 years, impaired extremities and unstable gait (in addition to muscle weakness) and were generally related to low motor function and mobility disorders, probably due to older age or an illness condition. Such patients would feel the need for self‐management at a much later stage of hospitalisation. However, this hypothesis needs to be explored in future studies.

### 4.7 Limitations of the study

This study was conducted over a limited period in a university hospital with mixed wards. The number of fall cases in each clinical department, or those related to specific diseases, was not separately analysed. Moreover, in this respect, the number of falls considered was inadequate, and a greater accumulation of cases can be hoped for in future studies, that should also evaluate the variables with significant differences in the Wilcoxon rank‐sum test. Additionally, validation using different cohorts in Japan and other countries would be necessary to validate the early and late risk variables found in this study.

## 5 CONCLUSIONS

In this study, early and late falls were categorised, and risk models were separately developed for each type of fall. The recognition of specific and common risk factors would together offer an opportunity to prevent falls during hospitalisation. As this concept would be offered to clinical practice to prevent falls in acute phase hospitals, it should be verified in future studies.

## RELEVANCE TO CLINICAL PRACTICE

Patient falls and related injuries are considered nursing‐sensitive indicators as fall prevention depends on the quality and quantity of nursing care (Heslop et al., 2014; Lucero et al., 2010). A systematic review of literature (Stalpers et al., 2015) showed that nurse staffing was inversely related to patient falls and that collaborative relationships between nurses and physicians, and nurse education and nursing experiences were important characteristics of the work environment. Fall prevention in hospitals was systematically reviewed by Hempel et al. (2013), who mentioned that although promising approaches exist, a better reporting of outcomes along with implementation, adherence, intervention components and comparison of group information is crucial for establishing evidence on how hospitals can successfully prevent falls.

Recently, several interesting papers that were published on this issue found that patients lack knowledge and understanding of falls, which leads to failure of preventive actions (de Freitas Luzia et al., 2020). Three focus group interviews with Australian hospitals also showed that most patients are unaware of the risk of falling during hospitalisation (Heng et al., 2021). The importance of implementing a multi‐component fall prevention programme by coordinating with patients for fall safety has been highlighted (Bargmann & Brundrett, 2020). Interestingly, a recent study has introduced a toolkit for preventing patient‐centred injuries and falls, and its efficacy was evaluated in a prospective study (Dykes et al., 2020). This was a nurse‐led fall prevention toolkit that was designed to link evidence‐based preventive interventions to patient‐specific fall risk factors and induce continuous engagement of patients and their families in the fall prevention process. The implementation resulted in a statistically significant 15% reduction in overall inpatient falls and 34% reduction in injurious falls; it also suggested possible tools for supporting patient engagement throughout hospitalisation in the fall prevention process. In line with these observations, it is necessary to identify patient‐specific risk factors (potential for early or late falls) and share these with patients, caregivers and medical staff for supporting patient engagement throughout hospitalisation.

This study describes sequential changes in risk factors for falls during hospitalisation in the acute care setting. Patients’ falls were divided into two groups based on the median of the fall date. The risk models developed for the two groups clearly characterised risk factors for them. The risk factors specific to early or late falls, or those common to both, were predicted, possibly leading to reasonable precautions to safeguard potential falls during the course of patients’ hospital stay.

## CONFLICTS OF INTEREST

None.

## AUTHOR CONTRIBUTIONS

MS, TS and TH contributed to study design. MS and TM performed data extraction and statistical analyses. All authors participated in drafting the article, editing and approving the final version of the article.

## Supporting information

Supplementary Material

Appendix S1

## Data Availability

The data are not publicly available due to privacy or ethical restrictions.
