# Supplementary material for: Risk stratification for early and late falls in acute care settings
Source: J Clin Nurs. 2022 Feb 27;32(3-4):494–505. doi: 10.1111/jocn.16267 (PMC10078671; doi:10.1111/jocn.16267)
Supplement: Supplementary file 1 — Supplementary Material [file JOCN-32-494-s002.docx]

**Supporting Information**

**Supplemental Table 1.** Risk models developed for late fallers among all patients (*n*=10,975) and among patients staying longer than 10 days (*n*=4,010)

**Supplemental Figure 1.** Occurrence of falls during hospitalization

Number of falls were plotted according to their occurrence. Falls were categorized into early (by day 10) and late (later than day 10) falls based on median day of falls (day 10).

**Supplemental Figure 2.** Receiver operating characteristic curve for late fallers among patients staying longer than 10 days

The 9 risk variables (Table3) were applied to data set including only patients staying hospital longer than 10 days (*n*=4,010). The Odds ratios were given as supplemental table 1. AUC was 0.776.

**Supplemental Table 1.** Risk models developed for late fallers among all patients (n=10,975) and among patients staying longer than 10 days (n=4,010)

| Variable Category | Variables | **Late falls** ^†^  **among all patients** | | | **Late falls** ^‡^  **among patients staying**  ≥ **10days** | | |
| --- | --- | --- | --- | --- | --- | --- | --- |
|  |  | OR | (95% CI) |  | OR | (95% CI) |  |
| Age | > 65 | 1.88 | (1.04-3.39) | * | 1.92 | (1,15-3.19) | * |
| History | fall history | 1.04 | (0.54-1.99) |  | 0.95 | (0.54-1.66) |  |
| Motor function | impaired extremities | 2.58 | (1.40-4.76) | ** | 2.76 | (1.67-4.56) | ** |
| Mobility | muscle weakness | 2.50 | (1.36-4.61) | ** | 1.84 | (1.10-3.11) | * |
|  | requiring mobility assistance | 1.81 | (0.95-3.43) |  | 1.50 | (0.87-2.57) |  |
|  | unstable gait | 2.88 | (1.49-5.54) | ** | 2.69 | (1.54-4.69) | ** |
| Cognition | impaired understanding | 0.72 | (0.30-1.70) |  | 0.76 | (0.38-1.54) |  |
| Medication | psychotropics | 1.73 | (0.96-3.12) |  | 1.86 | (1.10-3.13) | * |
| Personality | “do everything on one's own” | 0.74 | (0.22-2.49) |  | 0.83 | (0.32-2.15) |  |

*Notes*: OR = odds ratio; 95% CI = 95% confidence interval. † = C-index of development cohort 0.801 (Table 3), ‡ = C-index 0.776.

** *p*<0.01, * *p* <0.05.


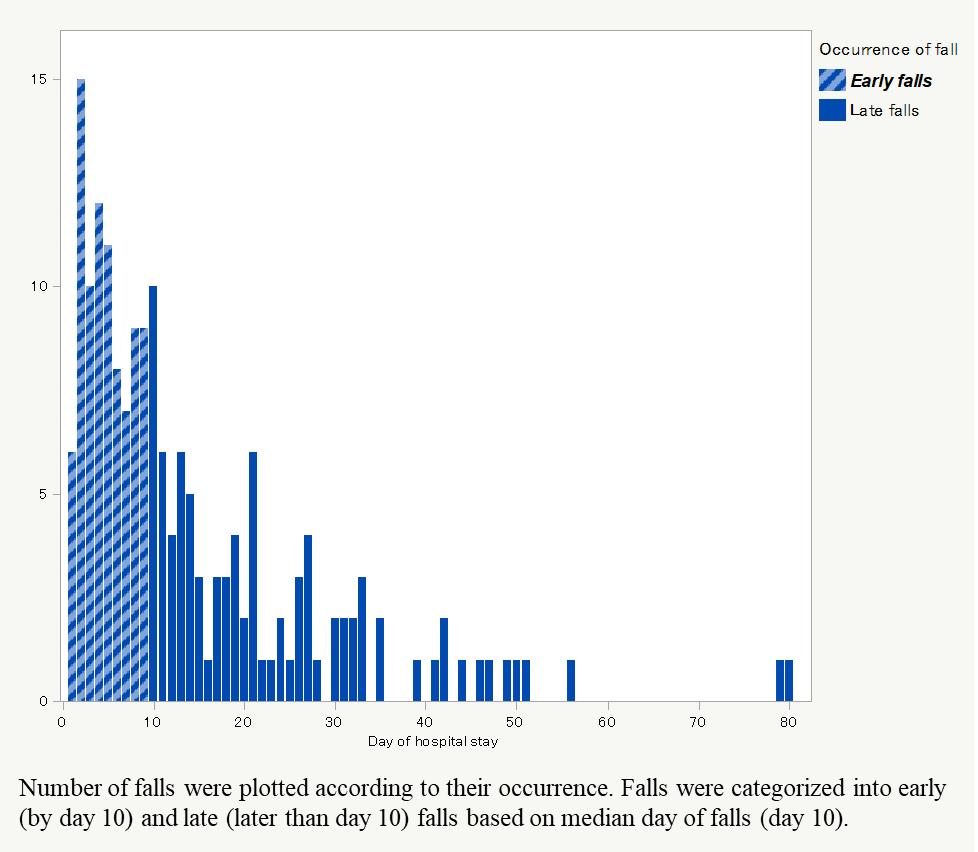


**Supplemental Figure 1.** Occurrence of falls during hospitalization


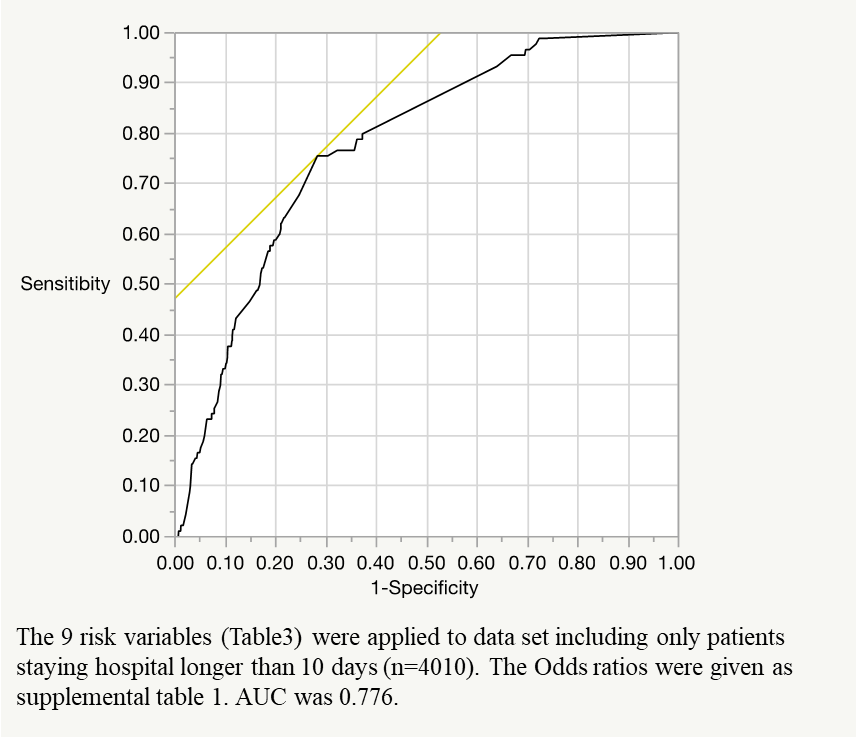


**Supplemental Figure 2.** Receiver operating characteristic curve for late fallers among patients staying longer than 10 days
